# The Effect of Financial Strain on the Health Outcomes of Older Mexican-Origin Adults: Findings From the Hispanic Established Population for the Epidemiological Study of the Elderly (H-EPESE)

**DOI:** 10.1177/00914150241231187

**Published:** 2024-02-14

**Authors:** Monica Hernandez, Kyriakos K. Markides, Philip Cantu

**Affiliations:** 1 Department of Population Health and Health Disparities, 12338 University of Texas Medical Branch Galveston, Galveston, TX, USA; 2 Internal Medicine-Geriatrics, 12338 University of Texas Medical Branch Galveston, Galveston, TX, USA

**Keywords:** financial strain, cumulative disadvantage, Hispanic adults, aging, health

## Abstract

Predictors of health across the life-course do not maintain the same significance in very late life and the role of financial strain in health outcomes of very old adults remain unclear. Data from adults aged 74  +  in waves 5 and 7 of the Hispanic Established Population for the Epidemiological Study of the Elderly (n  =  772) study was used to evaluate the role of financial strain on the health of older Mexican Americans who have the highest poverty rate of any racial or ethnic group in the United States. We evaluate the association between episodic (one wave) and persistent financial strain (two waves), with follow-up health outcomes (self-rated health, ADL (limitations in activities of daily living)/IADL (limitations in instrumental activities of daily living) disability, and depressive symptoms). Adults with persistent strain were twice as likely to experience depressive symptoms and three times more likely to experience IADL limitations than the unstrained. Our findings highlight the role of stress proliferation and allostatic load processes leading to deteriorated health over time.

## Introduction

Prior research shows that limited socioeconomic (socioeconomic status, SES) resources, such as food, medical care, education, and housing, propel racial and ethnic minorities with low SES toward poor health and mortality (Braudt et al., 2019; Karanth et al., 2019; National Academies of Sciences, Engineering, and Medicine et al., 2018; Sangaramoorthy et al., 2022). Income is a widely used SES proxy that can capture general access to health-related resources like food, healthcare, and the environment. Income typically can also capture current or past income at an individual and/or household level (Oakes & Kaufman, 2017).

Despite its widespread use in health research, obtaining income information is highly sensitive and subject to nonresponse bias (Roberts et al., 2020). Demands on income also widely vary according to family size, number of dependents, where and how people live, individual aspirations, and access to material resources other than income (Kahn & Pearlin, 2006). Moreover, income is not an ideal measure of SES among very old or retired adults who experience declining annual incomes and spending postretirement (Ebrahimi, 2019b, 2019a). Among this group, income may not reflect wealth accumulated over the lifespan including home equity, savings accounts, bonds and certificates of deposit, common stocks and mutual funds, annuities and cash-value insurance policies, and rental income.

Income information is also unable to capture subjective measures of financial wellbeing such as “financial strain” or stress/satisfaction related to finances (Friedline et al., 2021; Hertz-Palmor et al., 2021). Studies of financial strain typically ask about having enough money to make ends meet at the end of the month (Shippee et al., 2012). Such studies reveal that individuals in similar income brackets report varying levels of stress and financial strain according to their ability to adjust to their financial situation or material conditions (French & Vigne, 2019). Related explanations such as the *hedonistic treadmill* mentality have surfaced to help explain racial variations in perceptions of financial strain such that Black adults may be more likely than their White counterparts to perceive financial hardship as a normative state of being (Kahn & Pearlin, 2006).

Although there is a potential for reverse causation, studies have consistently linked financial strain to poor mental and physical health (Hertz-Palmor et al., 2021; Sharp et al., 2013; Steptoe et al., 2020). Studies have also documented health inequalities among adults with varying SES levels (i.e., the “social health gradient”) and respective *psychosocial* and *neomaterial* pathways have surfaced to help explain such trends (Singh et al., 2016). For example, the *psychosocial* pathway is explained by stress associated with perceptions of social disadvantage, in turn, increasing the risk of unhealthy behaviors and chronic health burden known as “allostatic load” (Gruenewald et al., 2012; McEwen, 1998). Conversely, the *neomaterial* pathway is explained by direct material disadvantage and health damaging exposures leading to inadequate access to material resources, such as medical care, food, and safe environments. While both pathways can help explain the social health gradient, polarization exists on the distinct or pre-eminent role of a single pathway on individual health outcomes (Moor et al., 2017; Skalická et al., 2009).

Life course studies on the social health gradient have also found that poor health is more likely among older individuals with chronic financial strain. For example, Kahn and Pearlin (2006) conducted interviews with a group of Medicare beneficiaries to examine the influence of the timing and persistence of financial hardship on physical and mental health outcomes over the life course. They asked beneficiaries to describe the level of financial hardship experienced during childhood (under age 18), early adulthood (ages 18–35), early middle age (ages 35–50), and later middle age (ages 50–65). Overall, Kahn and Pearlin (2006) found that persistent or cumulative financial hardship had more of a deleterious health effect than episodic financial hardship, particularly for hardships experienced during early adulthood and middle age. Such findings align with the theory of cumulative disadvantage (CAD) positing that population-level variation springs from systematic differences in given characteristics over time (e.g., money, health, and status) such that the “rich get richer and the poor get poorer” (Dannefer, 1987, 2003; O’Rand, 1996). Divergent socioeconomic inequalities overtime may also amplify the stress proliferation process, whereby stressors in late life can both persist and proliferate from stressors in early life across various life domains including work, personal, family, marriage, etc. (Pearlin, 1989; Pearlin et al., 2005).

CAD theory may also be applied to observed racial and ethnic health differences over the life course (Choi et al., 2017; Peterson, 2019; Zhang et al., 2016). For example, in the same study by Kahn and Pearlin (2006), Black adults were more likely than White adults to report two periods of financial hardship over the life course. Despite these findings, the health effect of persistent financial hardship on health was in fact stronger for White adults. The authors attributed these findings to a potential *survivorship bias* in which surviving Black adults were more resistant than their White counterparts to the health effects of financial hardship or, alternatively, were more likely to display the *hedonistic treadmill* mentality (Kahn & Pearlin, 2006).

The relationship between SES and psychological distress among older adult minorities is well-established in the extant literature (Bartholomae & Fox, 2022; Boen & Hummer, 2019; Brown et al., 2020; Gallo et al., 2013). Less established is the role of financial strain on the overall health of older Hispanic adults over the life course. Relative to other underrepresented groups, the health of older Hispanic adults is important to study in the context of financial strain due to their greater burden of chronic disease and limited availability of financial and health resources at older ages including lower social security income and other forms of retirement income replacing labor market wages (Ebrahimi, 2019b; Federal Interagency Forum on Aging-Related Statistics, 2020; Purcell, 2012). Moreover, older Hispanics are projected to be the oldest ethnic minority group by 2030 and the fastest growing by 2050, with Hispanic adults over 85 years old as the fastest growing subgroup relative to non-Hispanic Whites and any other racial/ethnic group (US Census Bureau, 2017). Such demographic shifts are projected to affect society as older Hispanic adults aged 65  +  have the highest poverty rate of any racial or ethnic group and account for a relatively large share of public programs such as Medicare and Social Security (Li & Dalaker, 2022; US Census Bureau, 2017).

The health of older Hispanic adults is also important to study in the context of financial strain due to epidemiological findings such as the *Hispanic Paradox* positing that Hispanic adults display favorable mortality profiles more similar to that of non-Hispanic White adults despite being more similar socioeconomically to Black adults (Markides & Coreil, 1986). Consistent explanations for the paradox have pointed to a *healthy migrant* effect suggesting that migrants are generally healthier than native-born individuals in the host country, or a *barrio advantage* suggesting that neighborhood health benefits may arise from increased labor force participation, intact family structures, home ownership, residential stability and low levels of discrimination (Markides & Rote, 2019; Martinez et al., 2015; Valles, 2016).

The literature clearly demonstrates nuances and heterogeneity in the Hispanic paradox among Hispanic subgroups. For example, in a study by Borrell and Lancet (2012), mortality advantages were observed among Hispanic women, but not men, as well as Mexican American and Central and South American women aged 25 to 44 years, Cuban women aged 45 to 64 years, and Puerto Rican and Mexican American women aged 65 years and older. Plausible explanations for this advantage were not noted; however the authors underscored the importance of closely examining nuances in Hispanic paradox by subgroup, age and gender (Borrell & Lancet, 2012). Related studies suggest that acculturation and SES play a large role in explaining the observed heterogeneity in the Hispanic paradox (Markides & Rote, 2019). Therefore, recognizing that significant demographic and socioeconomic diversity exists among Hispanics, other social comparison processes over the life course such as cumulative disadvantage may help explain the Hispanic Paradox, however studies have yet to explore such associations (Kim et al., 2019).

In this analysis, we examined the relationship between financial strain and physical and mental health outcomes among older Hispanic adults, specifically to evaluate the role of episodic versus persistent strain on health outcomes over time. We hypothesized that older adults with any level of financial strain would exhibit poorer health outcomes compared to adults with no financial strain due to perceived and/or actual material disadvantage leading to poor health, as per respective psychosocial and neomaterial processes. We further hypothesized that poor health would be greatest among financially strained adults undergoing persistent versus episodic strain, as per the CAD theory of poorer health among adults with cumulative financial strain over the life course.

## Methods

### Data

We used data from waves 5 (2004–2005) and 7 (2010–2011) of the Hispanic Established Population for the Epidemiologic Study of the Elderly (H-EPESE). H-EPESE is a longitudinal cohort-based study of noninstitutionalized Mexican Americans aged 65 and older from five southwestern states: Texas, New Mexico, Colorado, Arizona, and California (Cantu & Markides, 2019). H-EPESE respondents are sampled using a multistage area probability sampling design to be representative of 85% of the older Mexican Americans population. At baseline, 3,050 Mexican American participants aged 65 and older were initially sampled in 1993–1994 and were later followed up in 1995–1996 (N  =  2,438), 1998–1999 (N  =  1,980), 2000–2001 (N  =  1,685), and 2004–2005 (N  =  1,167). A probability sample of 902 Mexican American participants aged 75 and older were also added in 2004–2005 for a combined sample of N  =  2,069 in wave 5. This sample was followed up in 2007 and 2010 when participants were aged 80 and older in waves 6 (N  =  1,542) and 7 (N  =  1,078), respectively (Cantu & Markides, 2019). Our final analytical sample (N  =  772) was restricted to eligible participants with nonmissing predictor, outcome, or covariate data in both waves 5 and 7. See the final sample selection criteria flowchart in Appendix 1.

### Conceptual Model

**Figure 1.**
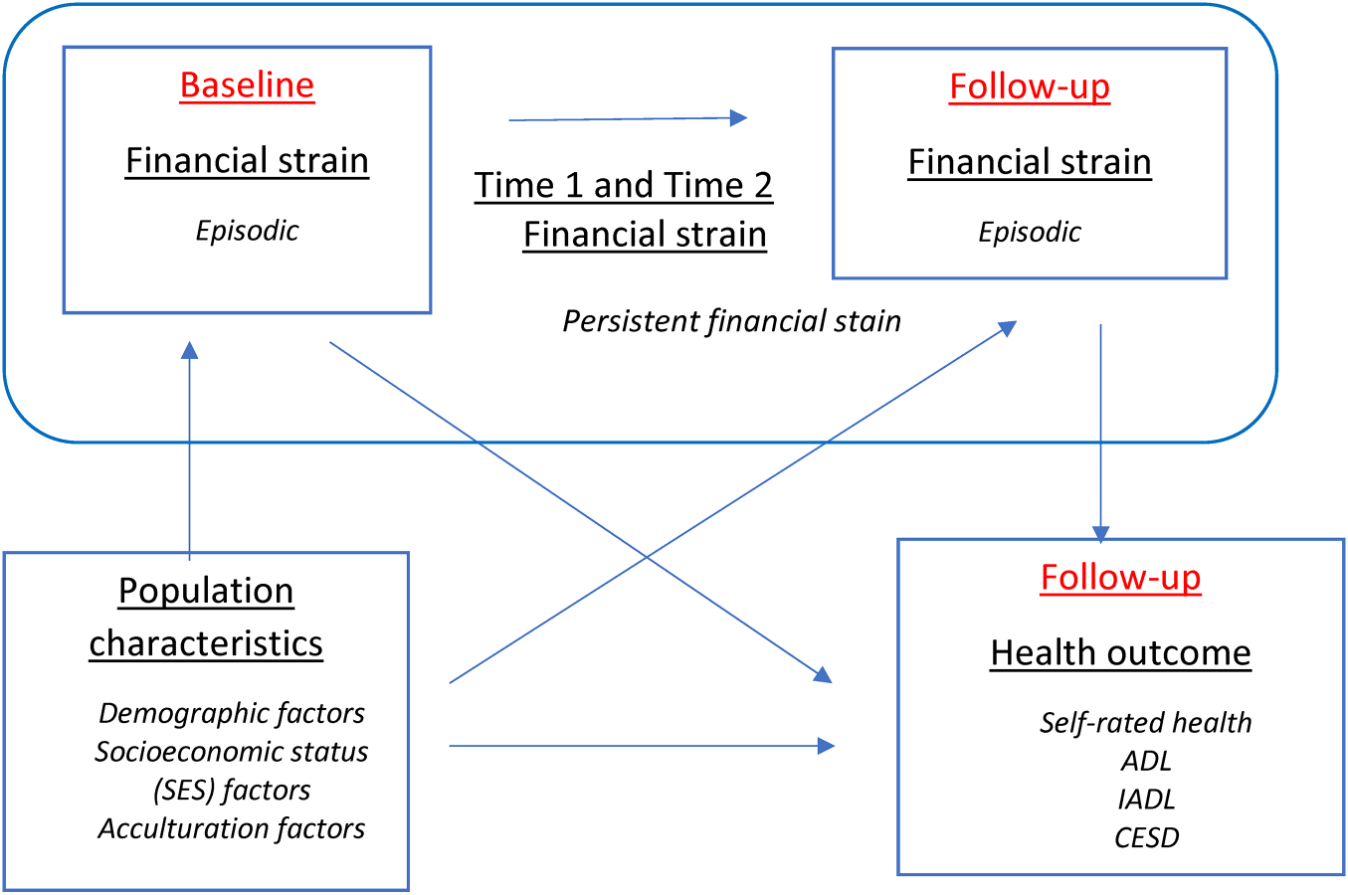
Conceptual model.

Our conceptual model (Figure 1) illustrates the mechanism through which persistent and episodic financial strain act on older adult health outcomes. Wave 5 is conceptualized as the *baseline wave* whereas wave 7 is conceptualized as the *follow-up wave*. Older adults can experience financial strain at neither, either (episodic strain), or both waves (persistent strain). These experiences of financial strain are contextualized by baseline demographic, SES, and acculturation factors associated with health in the follow-up wave. We expected that, compared to adults experiencing episodic strain, those experiencing persistent strain would display worse health in the follow-up wave.

### Variables

#### Key Exposure

Financial strain was initially measured by responses on two questions:

- *How much difficulty do you have in meeting monthly payments on your bills*? Options include (i) a great deal, (ii) some, (iii), a little, and (iv) none.
- *At the end of the month, do you usually end up with some money left over, just enough to make ends meet, or not enough money to make ends meet*? Options include (i) some money left over, (ii) just enough to make ends meet, and (iii) not enough money to make ends meet.

High financial strain was defined as responding either “a great deal” to having difficulty with monthly payments *or* “not enough money to make ends meet” at the end of the month. All other responses were coded as low financial strain including no strain. Two-wave financial strain was then coded as a four-level categorical variable to capture high financial strain across respective waves 5 and 7 as follows: (i) no strain, (ii) persistent strain, (iii) strain in the baseline wave only, and (iv) strain in the follow-up wave only. Missing responses as well as those indicating “don’t know” or “refused” in either of the two waves were coded as missing (n  =  2,051).

#### Key Outcomes

We assessed the effect of financial strain on four key health outcomes: self-rated health, ADL (limitations in activities of daily living), IADL (limitations in instrumental activities of daily living), and CES-D (Center for Epidemiologic Studies Depression Scale)*.* All variables were coded as a 0/1 dichotomous variable where 1 was defined as the poor health outcome of interest.

*Self-rated health* was identified by asking respondents how they would rate their overall health. Response options included (i) poor, (ii) fair, (iii) good, or (iv) excellent. We coded self-rated health as a 0/1 dichotomous variable: “good/excellent” health versus “poor/fair” health, respectively (Idler et al., 2004).

*ADL* and *IADL* were the two functional mobility variables assessed in this analysis. ADL was coded as a 0/1 dichotomous variable: “needing help” versus “not needing help” *with at least one ADL* personal care task, including bathing, grooming, dressing, walking, transferring, eating, and using the toilet (Katz et al., 1963). IADL was also coded as a 0/1 dichotomous variable: “needing help” versus “not needing help” *with at least one IADL,* including meal preparation, driving, balancing a checkbook, light housework, taking medications, shopping, and telephone communication (Bottari et al., 2010).

*Depressive symptoms* were examined with the CES-D. The scale is comprised of 20 questions asking how often in the past week respondents experienced symptoms associated with depression, such as restless sleep, difficulty focusing, and feeling sad. Response options range from 0 to 3 for each item (0  =  rarely, 1  =  some of the time, 2  =  much of the time, 3  =  most of the time). Scores range from 0 to 60 and are dichotomously coded as 0/1; a score of below 16 indicates low depressive symptoms whereas 16  +  indicates high depressive symptoms (Radloff, 1977).

*Covariates*: Our model covariates were guided by the literature on financial strain and health trajectories among older adults. We assessed the following the baseline covariates: age (74–84/85 + ), gender (male/female), marital status (married/not married), annual household income (under $10 K, $10K–19,999, $20K + ), education (under 6/6  +  years), health insurance coverage (Y/N), age of migration (US born, 0–19, 20–49, 50  +  years), and language of interview (English/Spanish).

We began our analyses by describing the baseline characteristics of older adults by level of financial strain (Table 1). We then used logistic regression models to evaluate the association between level of financial strain and health outcomes in the follow-up wave (Table 2). All regression models adjusted for baseline covariates and set the healthier group as the reference category for each variable. We reported odds ratios (OR) and 95% confidence intervals. Statistically significant *p*-values were determined as follows: *p ≤ .05, **p ≤ .01, and ***p ≤ .001.

**Table 1.** Descriptive Statistics by Financial Strain at the Baseline and Follow-Up Waves, Respectively Waves 5 and 7 (%).

Variable	No strain N = 476, 61.66%	Persistent strain N = 77, 9.97%	Strain in baseline wave only N = 146, 18.65%	Strain in follow-up wave only N = 76, 9.72%	Total N = 772, 100%	*p*-value
**Self-rated health, the follow-up wave**						***
Good/excellent self-rated health	38.66%	19.48%	28.47%	25.33%	33.55%	
Fair/poor self-rated health	61.34%	80.52%	71.53%	74.67%	66.45%	
**ADL limitation(s), the follow-up wave**						**
No ADL limitation(s)	60.29%	40.26%	50.69%	49.33%	55.44%	
1 + ADL limitation(s)	39.71%	59.74%	49.31%	50.67%	44.56%	
**IADL limitation(s), the follow-up wave**						***
No IADL limitation(s)	22.48%	6.49%	11.81%	10.67%	17.75%	
1 + IADL limitation(s)	77.52%	93.51%	88.19%	89.33%	82.25%	
**Depressive symptoms, the follow-up wave**						***
Low depressive symptoms	81.93%	59.74%	68.75%	64.00%	75.52%	
High depressive symptoms	18.07%	40.26%	31.25%	36.00%	24.48%	
**Age (years)**						*
74–84	84.66%	89.61%	78.47%	90.67%	84.59%	
85–109	15.34%	10.39%	21.53%	9.33%	15.41%	
*Mean (SD)*	*80.35 (3.86)*	*79.68 (3.59)*	*81.06 (3.88)*	*79.69 (3.21)*	*80.35 (3.80)*	
**Gender**						
Male	38.45%	35.06%	33.33%	34.67%	36.79%	
Female	61.55%	64.94%	66.67%	65.33%	63.21%	
**Marital status**						
Not married	51.05%	49.35%	59.72%	54.67%	52.85%	
Married	48.95%	50.65%	40.28%	45.33%	47.15%	
**Annual household income**						***
Under $10K	38.87%	70.13%	59.03%	49.33%	46.76%	
$10K–19,999	40.76%	25.97%	38.19%	38.67%	38.60%	
$20 K +	20.38%	3.90%	2.78%	12.00%	14.64%	
**Education**						***
Low education (under 6 years)	49.58%	83.12%	71.53%	58.67%	57.90%	
Some education (6 + years)	50.42%	16.88%	28.47%	41.33%	42.10%	
**Health insurance coverage**						
No	2.10%	3.90%	2.08%	2.67%	2.33%	
Yes	97.90%	96.10%	97.92%	97.33%	97.67%	
**Age of migration**						*
US born	58.61%	48.05%	53.47%	48.00%	55.57%	
0–19 years	6.72%	16.88%	3.47%	10.67%	7.51%	
20–49 years	27.10%	25.97%	33.33%	28.00%	28.24%	
50 + years	7.56%	9.09%	9.72%	13.33%	8.68%	
**Language of interview**						
English	21.22%	12.99%	18.06%	16.00%	19.30%	
Spanish	78.78%	87.01%	81.94%	84.00%	80.70%	
**Self-rated health, baseline**						**
Good/excellent self-rated health	45.38%	28.57%	36.11%	28.00%	40.28%	
Fair/poor self-rated health	54.62%	71.43%	64.89%	72.00%	59.72%	
**ADL limitation(s), baseline**						***
No ADL limitation(s)	81.93%	66.23%	69.44%	81.33%	77.98%	
1 + ADL limitation(s)	18.07%	33.77%	30.56%	18.67%	22.02%	
**IADL limitation(s), baseline**						***
No IADL limitation(s)	42.86%	28.57%	27.78%	26.67%	37.05%	
1 + IADL limitation(s)	57.14%	71.43%	72.22%	73.33%	62.95%	
**Depressive symptoms, baseline**						***
Low depressive symptoms	89.92%	77.92%	75.69%	76.00%	84.72%	
High depressive symptoms	10.08%	22.08%	24.31%	24.00%	15.28%	

*p ≤ .05, **p ≤ .01, ***p ≤ .001 using chi-squared/ANOVA test. ADL=limitations in activities of daily living; ANOVA=analysis of variance; IADL=limitations in instrumental activities of daily living.

**Table 2.** Adjusted Associations of Financial Strain Level and Other Covariates Predicting the Likelihood of Poor Health in the Follow-up Wave, Respectively Waves 5 and 7 (OR, 95%CI).

Variable	Fair/poor self-rated health n = 772	ADL limitation(s) n = 772	IADL limitation(s) n = 772	High depressive symptoms n = 772
**Financial strain**				
No strain (ref)				
Persistent strain	1.91* (1.00, 3.65)	1.75 * (1.01, 3.01)	2.92* (1.07, 7.99)	2.44** (1.39, 4.28)
Strain in baseline wave only	1.31 (0.84, 2.03)	1.09 (0.72, 1.66)	1.44 (0.78, 2.68)	1.60* (1.01, 2.54)
Strain in follow-up wave only	1.44 (0.81, 2.58)	1.66 (0.99, 2.81)	2.09 (0.91, 4.81)	2.16** (1.24, 3.77)
**Age (years)**				
74–84 (ref)				
85–109	0.70 (0.45, 1.09)	2.90*** (1.85, 4.55)	5.01*** (1.90, 13.15)	1.35 (0.84, 2.17)
**Gender**				
Male (ref)				
Female	0.95 (0.66, 1.37)	1.69* (1.18, 2.41)	2.18*** (1.38, 3.45)	1.60* (1.06, 2.43)
**Marital status**				
Not married (ref)				
Married	1.30 (0.89, 1.90)	1.04 (0.72, 1.49)	1.04 (0.64, 1.68)	0.77 (0.51, 1.16)
**Annual household income**				
$0–9,999	1.60 (0.96, 2.68)	1.56 (0.92, 2.64)	1.85 (0.97, 3.53)	0.92 (0.49, 1.70)
$10K–19,999	1.40 (0.86, 2.27)	1.42 (0.86, 2.35)	1.01 (0.58, 1.78)	1.01 (0.56, 1.85)
$20 K + (ref)				
**Education**				
Low education: 0–5 years	1.22 (0.86, 1.76)	1.33 (0.94, 1.89)	1.34 (0.83, 2.16)	1.39 (0.92, 2.08)
Some education: 6 + years (ref)				
**Health insurance coverage**				
No	0.42 (0.15, 1.16)	0.87 (0.32, 2.40)	3.05 (0.35, 26.51)	0.70 (0.21, 2.34)
Yes (ref)				
**Age of migration**				
US born (ref)				
0–19 years	0.92 (0.49, 1.75)	1.42 (0.77, 2.61)	1.30 (0.49, 3.45)	1.63 (0.86, 3.10)
20–49 years	1.26 (0.85, 1.88)	0.89 (0.61, 1.31)	0.84 (0.51, 1.39)	1.00 (0.65, 1.52)
50 + years	1.57 (0.80, 3.08)	0.81 (0.45, 1.46)	1.34 (0.48, 3.75)	0.90 (0.47, 1.71)
**Language of interview**				
English (ref)				
Spanish	1.32 (0.87, 2.02)	0.85 (0.56, 1.31)	1.06 (0.61, 1.84)	2.22** (1.26, 3.87)
**Self-rated health, baseline**				
Good/excellent self-rated health (ref)				
Fair/poor self-rated health	3.02*** (2.19, 4.18)	—	—	—
**ADL limitation(s), baseline**				
No ADL limitation(s) (ref)				
1 + ADL limitation(s)	—	3.48*** (2.36, 5.14)	—	—
**IADL limitation(s), baseline**				
No IADL limitation(s) (ref)				
1 + IADL limitation(s)	—	—	4.66*** (2.98, 7.28)	—
**Depressive symptoms, baseline**				
Low depressive symptoms (ref)				
High depressive symptoms	—	—	—	2.85*** (1.82, 4.47)

*p ≤ .05, **p ≤ .01, ***p ≤ .001. 95% CI= 95% confidence interval; ADL=limitations in activities of daily living; IADL=limitations in instrumental activities of daily living; OR=odds ratio.

## Results

Table 1 presents descriptive statistics, overall and by level of financial strain. The mean age of the total sample (n  =  772) was 80 years in the baseline wave; 63% were women, 85% had household incomes below $20 K, 98% had health insurance coverage, and 81% completed the interview in Spanish. Most adults in our sample reported no financial strain in either the baseline or follow-up wave (61.66%), whereas a minority reported persistent strain in both waves (9.97%). Across the follow-up wave health outcomes, most adults reported fair/poor self-rated health (66.45%), no ADL limitations (55.44%), 1 + IADL limitation (82.25%), and low depressive symptoms (75.52%). Adults with persistent strain were more likely than adults without strain to be aged between 74 and 84 (89.61% vs. 84.66%), be female (64.94% vs. 61.55%), have annual household incomes under $10 K (70.13% vs. 38.87%), and have less than 6 years of education (83.12% vs. 49.58%). Significant differences between the financial strain categories were observed for age and age of migration (*p* < .05); the follow-up wave ADL disability (*p* < .01); and the follow-up wave self-rated health, IADL disability, depressive symptoms, annual household income, and education (*p* < .001).

Table 2 presents results from logistic regression analysis evaluating the association between level of financial strain on incident poor health in the follow-up wave, controlling for the baseline wave covariates. Our findings indicate that adults with episodic strain in either the baseline or follow-up wave were up to two times more likely than adults with no strain to experience poor health across all health outcomes (1.09 < OR < 2.16); however, overall odds of poor health were greater among adults with strain in the follow-up wave versus the baseline wave only (respectively, 1.44 < OR < 2.16 vs. 1.09 < OR < 1.60). Moreover, adults with persistent strain in the baseline or follow-up wave were up to two times more likely than those without strain to report poor self-rated health, ADL limitations, and depressive symptoms (respectively, OR = 1.91 [1.00, 3.65]; OR = 1.75 [1.01, 3.01]; OR = 2.44 [1.39, 4.28]) and three times more likely to experience IADL limitations (OR = 2.92 [1.07, 7.99]). The overall odds of poor health was also greater among those with persistent strain (1.75 < OR < 2.92) versus episodic strain in either the baseline or follow-up wave (respectively, 1.09 < OR < 1.60 vs. 1.44 < OR < 2.16). Other key drivers of poor health in our analysis were age, gender, and language, with the oldest old (aged 85 + ), females, and individuals who completed an interview in Spanish reporting a significantly greater odds of ADL/IADL limitations and depressive symptoms than their respectively younger, male and English-speaking counterparts.

## Discussion

In this analysis, we examined the relationship between the level of financial strain and poor health among older Hispanic adults, specifically the role of episodic versus persistent strain on health outcomes over time. Both our hypotheses were supported as we found overall evidence for both the persistent effect *and* episodic effect of financial strain on health outcomes, with persistent strain posing a greater health threat than episodic strain at baseline and follow-up waves. Likely explanations from the literature include *the stress proliferation process*, whereby a “spillover” of stressors from early to late life (i.e., work, personal, family, marriage, etc.) erodes personal resources including self-efficacy and coping mechanisms, in turn, leading to subsequent *allostatic load* or disrupted biological systems and deteriorated health over time (McEwen, 1998; Pearlin, 1989; Pearlin et al., 1997, 2005).

Compared to individuals without any strain, we found a relatively greater odds of poor health among individuals with persistent versus episodic strain with a greater odds of poor health among adults with episodic strain in the follow-up versus baseline wave. These findings align well with the Kahn and Pearlin (2006) study that found worse health among older adults aged 65  +  with persistent versus episodic strain and even more so among adults who experienced strain between 50 and 65 years versus 35 and 50 years. The authors explained that ageing adults are likely to experience a greater health impact of financial strain due to higher number of diagnosed health conditions and functional capabilities over time. We agree with this explanation and further suggest that poorer health among ageing adults with strain in follow-up (2010–2011) versus baseline waves (2004–2005) in our analysis might be explained by the unobserved effects of the 2008 Recession; a period in which widespread unemployment precipitated insurance, income, and wealth decline among 14 million American adults, most notably among racial and ethnic minorities and persons of low SES (Herbert & Apgar, 2010; Johnson, 2009; Reid & Laderman, 2009; US Bureau of Labor Statistics (BLS), 2012).

We report similar findings to the Angel et al. (2003) study in which Mexican-origin older adults aged 65  +  with high financial strain were more likely than their unstrained counterparts to experience worse physical and mental health outcomes. Angel et al. (2003) also found reverse associations on the influence of health indicators on subsequent financial strain. The authors proposed this “demoralization package” could impair an individual's sense of control in key life aspects, in turn, increasing feelings of financial strain (Angel et al., 2003). We suggest this demoralization package could also work bidirectionally among ageing adults given our findings of baseline financial strain influencing health outcomes in the follow-up wave as well as literature on increased stress associated with perceptions of social disadvantage (i.e., psychosocial pathway).

Our findings also align with results from Shippee et al.’s (2012) study of an older sample of non-Hispanic Black and White women from the National Longitudinal Survey of Mature Women (NLSM) aged 66 to 80 years. Although men were not included in the study, Shippee et al. reported a higher likelihood of poor health outcomes among women with persistent financial strain, as was also found in our analysis. Moreover, the likelihood of poor self-rated health in the study was nearly two times higher among women reporting 4  +  versus 1 episode of financial strain (Shippee et al., 2012). The authors also highlighted the importance of cumulative inequality in explaining their results; citing the distinctly higher risk of financial strain among women due to their position in the labor force, family roles, and lower earnings compared with men. Future research might therefore examine differences by gender and/or headship status in the relationship between chronic financial strain and poor health.

While annual household income had no statistically significant association with any of the studied health outcomes in our analysis, any level of financial strain had statistically significant associations with all health outcomes, most notably with depressive symptoms. This finding reinforces differences in the effects of psychosocial versus neomaterial pathways on individual health outcomes, as found in the literature (Moor et al., 2017; Singh et al., 2016; Skalická et al., 2009). Despite these differences, we acknowledge evidence of the dynamic interaction between the pathways and subsequent multiplicative health effects over the life course (Pearlin et al., 2005; Szanton et al., 2010). Pearlin et al. emphasize this point by proposing *the stress proliferation process,* whereby stressors in late life can both persist and proliferate from stressors in early life across various life domains (Pearlin, 1989; Pearlin et al., 2005).

Aside from financial strain, other key significant drivers of poor health in our analysis were age, gender, and language. The oldest old (aged 85 + ), females, and individuals who completed an interview in Spanish had significantly greater odds of ADL/IADL limitations and depressive symptoms than their respectively younger, male and English-speaking counterparts. This heterogeneity in health outcomes among different population subgroups has been attributed in the literature to differential socioeconomic and life course trajectories among older adults. House et al. (1990) suggested that survivorship bias, cohort-specific characteristics, or period effects could help explain socioeconomic health differences among ageing adults, along with varying economic and political contexts. They also suggested these differences would narrow among the older (aged 75 and older) and the oldest old (aged 85 and older) with the advent of retirement, federal income maintenance, and healthcare available to adults aged 65 and older (House et al., 1990). Nevertheless, as per Kahn and Pearlin (2006), higher costs associated with deteriorating health in old age may counteract this type of financial convergence in adults aged 75  +  years.

This analysis had several limitations. First, we only assessed two waves of data and might be able to better determine a stronger longitudinal effect of financial strain with more waves of data. Estimates from additional sensitivity analyses conducted on a shorter follow-up period between waves 5 and 6 were slightly smaller than our main results (see online Supplemental Table). For instance, in sensitivity analyses, slightly attenuated odds ratios were observed for persistent financial strain across all outcomes. Slightly attenuated odds ratios were also observed for follow-up financial strain on IADLs and depressive symptoms. Together these findings indicate the generally greater effects of persistent and follow-up financial strain on health outcomes over longer versus shorter follow-up periods. Second, we did not assess the population aged 65 to 73 due to sample size limitations; however, more waves of data might help increase the age variability in our sample. Third, because no meaningful longitudinal weights exist in the H-EPESE dataset, our unweighted results are generalizable only to U.S. Mexican American adults with similar characteristics to our study sample. Finally, small sample sizes from select categorical variables, including annual household income and health insurance coverage, may have limited our power and ability to detect statistical significance within all models.

## Conclusion

Persistent financial strain is more strongly associated with negative health than episodic strain, even when controlling for demographic and socioeconomic covariates. Our findings reinforce the multidimensionality of economic wellbeing and the subsequent importance of exploring the dynamic, multiplicative effects of psychosocial and neomaterial pathways linking financial strain to poor health over the life course. Relevant future research may examine differences in the health effects of financial strain by level of household income and other sociodemographic characteristics such as gender. Future research may also explore the extent to which the “double jeopardy” of age and race/ethnicity influences the health of financially strained older minorities relative to their younger, nonminority counterparts (Dowd & Bengtson, 1978; Tran et al., 1996). Moreover, related interventions may be tailored to address the effects of significant life course events on financial strain, for example, an intervention to help older adults improve their sense of control over complex financial decisions including refinancing a mortgage, managing investments, or retiring (Chan et al., 2002; Federal Reserve Board [FRB], 2020).

## Supplemental Material

sj-docx-1-ahd-10.1177_00914150241231187 - Supplemental material for The Effect of Financial Strain on the Health Outcomes of Older Mexican-Origin Adults: Findings From the Hispanic Established Population for the Epidemiological Study of the Elderly (H-EPESE) Supplemental material, sj-docx-1-ahd-10.1177_00914150241231187 for The Effect of Financial Strain on the Health Outcomes of Older Mexican-Origin Adults: Findings From the Hispanic Established Population for the Epidemiological Study of the Elderly (H-EPESE) by Monica Hernandez, Kyriakos K. Markides and Philip Cantu in The International Journal of Aging and Human Development

sj-docx-2-ahd-10.1177_00914150241231187 - Supplemental material for The Effect of Financial Strain on the Health Outcomes of Older Mexican-Origin Adults: Findings From the Hispanic Established Population for the Epidemiological Study of the Elderly (H-EPESE) Supplemental material, sj-docx-2-ahd-10.1177_00914150241231187 for The Effect of Financial Strain on the Health Outcomes of Older Mexican-Origin Adults: Findings From the Hispanic Established Population for the Epidemiological Study of the Elderly (H-EPESE) by Monica Hernandez, Kyriakos K. Markides and Philip Cantu in The International Journal of Aging and Human Development
