# Supplementary material for: The Effect of Financial Strain on the Health Outcomes of Older Mexican-Origin Adults: Findings From the Hispanic Established Population for the Epidemiological Study of the Elderly (H-EPESE)
Source: Int J Aging Hum Dev. 2024 Feb 14;99(1):3–24. doi: 10.1177/00914150241231187 (PMC11295423; doi:10.1177/00914150241231187)
Supplement: sj-docx-1-ahd-10.1177_00914150241231187 - Supplemental material for The Effect of Financial Strain on the Health Outcomes of Older Mexican-Origin Adults: Findings From the Hispanic Established Population for the Epidemiological Study of the Elderly (H-EPESE) [file sj-docx-1-ahd-10.1177_00914150241231187.docx]

**APPENDIX 1: Final sample selection criteria (HEPESE sample; Wave 5 and 7)**

Baseline sample

**(N= 2,945)**

Excluding respondents <65 years at wave 5 **(n=876)**

Respondents 65+ years at wave 5

**(N=2,069)**

Excluding missing wave 5 and 7 financial strain data **(n=1,175)**

Non-missing wave 5 and 7 financial strain data

**(N=894)**

Excluding missing wave 5 covariates:

**(n=103)**

Non-missing wave 5 covariates

**(N=791)**

Excluding missing wave 5 outcomes:

**(n=19)**

Non-missing wave 5 outcomes

**(N=772)**

Final sample

**(N= 772)**
