# Supplementary material for: The Effect of Financial Strain on the Health Outcomes of Older Mexican-Origin Adults: Findings From the Hispanic Established Population for the Epidemiological Study of the Elderly (H-EPESE)
Source: Int J Aging Hum Dev. 2024 Feb 14;99(1):3–24. doi: 10.1177/00914150241231187 (PMC11295423; doi:10.1177/00914150241231187)
Supplement: sj-docx-2-ahd-10.1177_00914150241231187 - Supplemental material for The Effect of Financial Strain on the Health Outcomes of Older Mexican-Origin Adults: Findings From the Hispanic Established Population for the Epidemiological Study of the Elderly (H-EPESE) [file sj-docx-2-ahd-10.1177_00914150241231187.docx]

**Sensitivity analysis (online supplement): Adjusted associations of financial strain level and other covariates predicting the likelihood of poor health in the follow-up wave, respectively Waves 6 and 7 (OR, 95%CI)**

| Variable | Fair/poor self-rated health  n=1,074 | ADL limitation(s)  n=1,074 | IADL limitation(s)  n=1,074 | High depressive symptoms  n= 1,074 |
| --- | --- | --- | --- | --- |
| Financial strain |  |  |  |  |
| No strain (ref) |  |  |  |  |
| Persistent strain | 1.76 (1.15, 2.68)** | 2.45 (1.61, 3.73)*** | 2.16 (1.28, 3.65)** | 2.14 (1.41, 3.23)*** |
| Strain in baseline wave only | 1.26 (0.83, 1.91) | 1.31 (0.85, 2.02) | 1.63 (0.98, 2.72) | 0.97 (0.61, 1.57) |
| Strain in follow-up wave only | 1.46 (0.97, 2.19) | 1.71 (1.13, 2.60)** | 0.66 (0.43, 1.03) | 1.71 (1.11, 2.64)* |
| Age (years) |  |  |  |  |
| 74 to 84 (ref) |  |  |  |  |
| 85 to 109 | 0.92 (0.66, 1.30) | 1.98 (1.37, 2.86)*** | 2.28 (1.43, 3.64)*** | 1.15 (0.78, 1.70) |
| Gender |  |  |  |  |
| Male (ref) |  |  |  |  |
| Female | 1.14 (0.84, 1.54) | 1.21 (0.88, 1.67) | 1.76 (1.25, 2.46)*** | 1.49 (1.04, 2.14)* |
| Marital Status |  |  |  |  |
| Not married (ref) |  |  |  |  |
| Married | 0.97 (0.71, 1.34) | 0.93 (0.67, 1.31) | 0.83 (0.58, 1.19) | 0.99 (0.68, 1.43) |
| Annual household income |  |  |  |  |
| $0-9,999 | 1.22 (0.78, 1.91) | 2.09 (1.26, 3.46)** | 1.30 (0.79, 2.13) | 3.02 (1.56, 5.83)*** |
| $10K-19,999 | 1.41 (0.93, 2.14) | 1.50 (0.92, 2.43) | 1.17 (0.74, 1.84) | 2.40 (1.26, 4.58)** |
| $20K +(ref) |  |  |  |  |
| Education |  |  |  |  |
| Low education: 0-5 years | 1.06 (0.79, 1.43) | 1.27 (0.93, 1.75) | 1.46 (1.04, 2.06)* | 1.09 (0.77, 1.55) |
| Some education: 6+ years (ref) |  |  |  |  |
| Health insurance coverage |  |  |  |  |
| No | 1.14 (0.45, 2.87) | 0.81 (0.31, 2.12) | 2.44 (0.66, 9.08) | 1.99 (0.79, 5.04) |
| Yes (ref) |  |  |  |  |
| Age of Migration |  |  |  |  |
| US born (ref) |  |  |  |  |
| 0 to 19 years | 0.72 (0.44, 1.19) | 0.69 (0.40, 1.19) | 0.69 (0.37, 1.28) | 1.02 (0.57, 1.84) |
| 20-49 years | 1.23 (0.88, 1.73) | 0.60 (0.42, 0.85)** | 0.58 (0.40, 0.85)** | 1.19 (0.81, 1.76) |
| 50+ years | 1.25 (0.74, 2.12) | 0.71 (0.42, 1.20) | 0.72 (0.40, 1.31) | 1.80 (1.08, 3.02)* |
| Language of interview |  |  |  |  |
| English (ref) |  |  |  |  |
| Spanish | 1.33 (0.93, 1.91) | 1.49 (1.00, 2.22)* | 1.17 (0.77, 1.79) | 0.75 (0.49, 1.16) |
| Self-rated health, baseline |  |  |  |  |
| Good/excellent self-rated health (ref) | 3.52 (2.69, 4.61)*** | - - | - - | - - |
| Fair/poor self-rated health |  |  |  |  |
| ADL limitation(s), baseline |  |  |  |  |
| No ADL limitation(s) (ref) | - - |  | - - | - - |
| 1+ ADL limitation(s) |  | 10.03 (6.91, 14.57)*** |  |  |
| IADL limitation(s), baseline |  |  |  |  |
| No IADL limitation(s) (ref) | - - | - - |  | - - |
| 1+ IADL limitation(s) |  |  | 5.88 (4.28, 8.07)*** |  |
| Depressive symptoms, baseline |  |  |  |  |
| Low depressive symptoms (ref) | - - | - - | - - |  |
| High depressive symptoms |  |  |  | 3.70 (2.56, 5.34)*** |

*p ≤ 0.05, **p ≤ 0.01, ***p ≤ 0.001
